# Supplementary material for: Comparative genomics provides new insights into the diversity, physiology, and sexuality of the only industrially exploited tremellomycete: Phaffia rhodozyma
Source: BMC Genomics. 2016 Nov 9;17:901. doi: 10.1186/s12864-016-3244-7 (PMC5103461; doi:10.1186/s12864-016-3244-7)
Supplement: Additional file 6: — List of orphan genes with links to PFAM (related to Additional file 1: Table S1). (ZIP 1428 kb) [file 12864_2016_3244_MOESM6_ESM.zip › PFAM_UNIQUE/G01210_P.html]

#                                                                            --- full sequence --- -------------- this domain -------------   hmm coord   ali coord   env coord
  
# target name        accession   tlen query name           accession   qlen   E-value  score  bias   #  of  c-Evalue  i-Evalue  score  bias  from    to  from    to  from    to  acc description of target
  
#------------------- ---------- ----- -------------------- ---------- ----- --------- ------ ----- --- --- --------- --------- ------ ----- ----- ----- ----- ----- ----- ----- ---- ---------------------
  
G01210\_P             -            260 AAA\_13               PF13166.1    712     0.054   10.6   1.3   1   1   0.00041     0.065   10.3   1.3   285   440    91   256    54   259 0.75 - AAA domain  
G01210\_P             -            260 ADIP                 PF11559.3    151       9.8    4.7   8.7   1   2     0.025       9.5    4.8   0.8    87   125    93   131    33   137 0.65 - Afadin- and alpha -actinin-Binding  
G01210\_P             -            260 ADIP                 PF11559.3    151       9.8    4.7   8.7   2   2     0.046        17    3.9   1.7    43    95   196   248   167   258 0.58 - Afadin- and alpha -actinin-Binding  
G01210\_P             -            260 APG6                 PF04111.7    315      0.37    8.4   7.3   1   2      0.11        19    2.8   0.1    26    85    69   132    37   140 0.64 - Autophagy protein Apg6  
G01210\_P             -            260 APG6                 PF04111.7    315      0.37    8.4   7.3   2   2    0.0033      0.58    7.8   3.1    23    92   187   254   174   259 0.79 - Autophagy protein Apg6  
G01210\_P             -            260 ATG16                PF08614.6    194       1.3    7.5   9.4   1   2   0.00068      0.17   10.4   1.4    35   141    27   132    11   137 0.84 - Autophagy protein 16 (ATG16)  
G01210\_P             -            260 ATG16                PF08614.6    194       1.3    7.5   9.4   2   2      0.28        69    1.9   1.3    30   118   197   235   156   259 0.47 - Autophagy protein 16 (ATG16)  
G01210\_P             -            260 BicD                 PF09730.4    717      0.37    7.7   7.8   1   2       0.1        23    1.8   0.1   285   319    99   133    24   140 0.72 - Microtubule-associated protein Bicaudal-D  
G01210\_P             -            260 BicD                 PF09730.4    717      0.37    7.7   7.8   2   2    0.0011      0.25    8.3   3.5    31    87   202   258   183   259 0.87 - Microtubule-associated protein Bicaudal-D  
G01210\_P             -            260 CALCOCO1             PF07888.6    546     0.061   10.3   5.6   1   1   0.00018     0.083    9.8   5.6   168   329    70   251    38   258 0.63 - Calcium binding and coiled-coil domain (CALCOCO1) like  
G01210\_P             -            260 CDC37\_N              PF03234.9    177      0.04   12.9   1.2   1   2     0.024        21    4.1   0.0    80   154    56   134    22   153 0.65 - Cdc37 N terminal kinase binding  
G01210\_P             -            260 CDC37\_N              PF03234.9    177      0.04   12.9   1.2   2   2    0.0018       1.6    7.7   0.3    20    75   181   234   175   255 0.80 - Cdc37 N terminal kinase binding  
G01210\_P             -            260 CENP-F\_leu\_zip       PF10473.4    140       2.6    6.6   5.7   1   2      0.52     1e+02    1.4   0.1    63    88   101   126    61   136 0.60 - Leucine-rich repeats of kinetochore protein Cenp-F/LEK1  
G01210\_P             -            260 CENP-F\_leu\_zip       PF10473.4    140       2.6    6.6   5.7   2   2     0.027       5.2    5.6   2.2    32    80   206   254   198   258 0.83 - Leucine-rich repeats of kinetochore protein Cenp-F/LEK1  
G01210\_P             -            260 CENP-Q               PF13094.1    160         5    5.8   9.6   1   2     0.077        24    3.6   0.6    32    74    91   133    89   137 0.86 - CENP-Q, a CENPA-CAD centromere complex subunit  
G01210\_P             -            260 CENP-Q               PF13094.1    160         5    5.8   9.6   2   2     0.012       3.8    6.2   1.9    36    81   200   245   184   257 0.83 - CENP-Q, a CENPA-CAD centromere complex subunit  
G01210\_P             -            260 CorA                 PF01544.13   292       1.6    6.3   6.7   1   2    0.0011      0.68    7.6   0.6   156   228    67   136    39   139 0.80 - CorA-like Mg2+ transporter protein  
G01210\_P             -            260 CorA                 PF01544.13   292       1.6    6.3   6.7   2   2     0.059        35    1.9   0.8   160   219   162   218   147   232 0.77 - CorA-like Mg2+ transporter protein  
G01210\_P             -            260 Cortex-I\_coil        PF09304.5    107       5.6    5.7  10.1   1   2    0.0033       1.8    7.3   0.3    37    71   104   138    89   140 0.82 - Cortexillin I, coiled coil  
G01210\_P             -            260 Cortex-I\_coil        PF09304.5    107       5.6    5.7  10.1   2   2      0.25   1.3e+02    1.3   4.3    36   104   179   251   150   255 0.72 - Cortexillin I, coiled coil  
G01210\_P             -            260 DUF3450              PF11932.3    252       2.1    6.2   5.1   1   3      0.27   3.2e+02   -0.9   0.1   106   126    62    82    24   104 0.77 - Protein of unknown function (DUF3450)  
G01210\_P             -            260 DUF3450              PF11932.3    252       2.1    6.2   5.1   2   3      0.13   1.5e+02    0.1   0.3    71   102    93   124    87   144 0.54 - Protein of unknown function (DUF3450)  
G01210\_P             -            260 DUF3450              PF11932.3    252       2.1    6.2   5.1   3   3    0.0012       1.4    6.8   0.4    19    85   188   254   159   258 0.73 - Protein of unknown function (DUF3450)  
G01210\_P             -            260 DUF4200              PF13863.1    126      0.84    8.3   7.1   1   2     0.024       7.5    5.2   0.1    67   106    93   132    61   135 0.82 - Domain of unknown function (DUF4200)  
G01210\_P             -            260 DUF4200              PF13863.1    126      0.84    8.3   7.1   2   2     0.062        20    3.8   3.9    26    90   192   256   179   258 0.74 - Domain of unknown function (DUF4200)  
G01210\_P             -            260 DUF641               PF04859.7    132      0.93    7.9   5.0   1   2    0.0028       5.6    5.4   0.1    70   120    82   132    57   137 0.79 - Plant protein of unknown function (DUF641)  
G01210\_P             -            260 DUF641               PF04859.7    132      0.93    7.9   5.0   2   2     0.011        23    3.4   1.5    68    99   192   223   154   255 0.55 - Plant protein of unknown function (DUF641)  
G01210\_P             -            260 EzrA                 PF06160.7    560       1.2    5.9   4.7   1   2    0.0086       5.7    3.6   0.1   108   152    93   137    85   140 0.79 - Septation ring formation regulator, EzrA  
G01210\_P             -            260 EzrA                 PF06160.7    560       1.2    5.9   4.7   2   2    0.0096       6.3    3.5   1.5    95   154   199   258   175   259 0.89 - Septation ring formation regulator, EzrA  
G01210\_P             -            260 Filament             PF00038.16   312     0.048   11.8   5.9   1   2      0.09        15    3.6   0.1   195   236    93   134    39   139 0.81 - Intermediate filament protein  
G01210\_P             -            260 Filament             PF00038.16   312     0.048   11.8   5.9   2   2    0.0012       0.2    9.7   2.1   235   297   178   240   165   257 0.90 - Intermediate filament protein  
G01210\_P             -            260 FliJ                 PF02050.11   123      0.34    9.7   7.0   1   2    0.0075         5    5.9   0.3     5    39    93   127    90   138 0.72 - Flagellar FliJ protein  
G01210\_P             -            260 FliJ                 PF02050.11   123      0.34    9.7   7.0   2   2    0.0055       3.6    6.3   1.7    12    75   198   256   183   259 0.86 - Flagellar FliJ protein  
G01210\_P             -            260 GAS                  PF13851.1    201       2.2    6.1   5.4   1   2     0.069        13    3.5   0.1   148   189    91   132    58   137 0.64 - Growth-arrest specific micro-tubule binding  
G01210\_P             -            260 GAS                  PF13851.1    201       2.2    6.1   5.4   2   2     0.061        12    3.7   1.9    78   128   204   254   178   259 0.62 - Growth-arrest specific micro-tubule binding  
G01210\_P             -            260 Hat1\_N               PF10394.4    161     0.014   14.1   0.1   1   2   6.6e-05       0.2   10.4   0.0    74   155    46   133    37   135 0.70 - Histone acetyl transferase HAT1 N-terminus  
G01210\_P             -            260 Hat1\_N               PF10394.4    161     0.014   14.1   0.1   2   2     0.036   1.1e+02    1.5   0.0    65   108   191   234   177   246 0.78 - Histone acetyl transferase HAT1 N-terminus  
G01210\_P             -            260 IncA                 PF04156.9    191      0.17   10.1  10.8   1   2    0.0019      0.19   10.0   0.6   122   187    86   133    19   139 0.61 - IncA protein  
G01210\_P             -            260 IncA                 PF04156.9    191      0.17   10.1  10.8   2   2     0.084       8.3    4.6   3.8    60   140   178   257   147   258 0.88 - IncA protein  
G01210\_P             -            260 MscS\_porin           PF12795.2    240       1.1    7.2   8.7   1   2      0.01       3.2    5.7   1.4    99   139    93   133    89   139 0.82 - Mechanosensitive ion channel porin domain  
G01210\_P             -            260 MscS\_porin           PF12795.2    240       1.1    7.2   8.7   2   2     0.012       3.6    5.5   1.0    17    85   186   254   176   258 0.58 - Mechanosensitive ion channel porin domain  
G01210\_P             -            260 Myosin\_tail\_1        PF01576.14   859      0.99    5.7   9.8   1   2    0.0096       1.4    5.2   0.2   331   437    21   132    16   138 0.77 - Myosin tail  
G01210\_P             -            260 Myosin\_tail\_1        PF01576.14   859      0.99    5.7   9.8   2   2     0.024       3.6    3.8   4.5   588   646   198   256   173   259 0.69 - Myosin tail  
G01210\_P             -            260 OmpH                 PF03938.9    158      0.26    9.9   7.2   1   2      0.47        83    1.7   0.1    45    74    95   124    56   136 0.78 - Outer membrane protein (OmpH-like)  
G01210\_P             -            260 OmpH                 PF03938.9    158      0.26    9.9   7.2   2   2     0.001      0.18   10.4   2.5    27   111   178   257   172   259 0.62 - Outer membrane protein (OmpH-like)  
G01210\_P             -            260 Phage\_mat-A          PF03863.8    400     0.044   11.5   0.0   1   1     9e-06     0.054   11.2   0.0    22   141    50   170    35   223 0.78 - Phage maturation protein  
G01210\_P             -            260 PhoU                 PF01895.14    88     0.053   12.7   2.0   1   2    0.0012       2.4    7.3   0.0    27    51    98   126    90   149 0.72 - PhoU domain  
G01210\_P             -            260 PhoU                 PF01895.14    88     0.053   12.7   2.0   2   2     0.013        25    4.1   0.6     6    51   176   221   172   257 0.70 - PhoU domain  
G01210\_P             -            260 Reo\_sigmaC           PF04582.7    326      0.21    9.4   6.7   1   3      0.73   1.5e+02    0.0   0.5    87   138     6    58     1    65 0.69 - Reovirus sigma C capsid protein  
G01210\_P             -            260 Reo\_sigmaC           PF04582.7    326      0.21    9.4   6.7   2   3    0.0016      0.34    8.7   3.8    40   161    64   186    44   193 0.70 - Reovirus sigma C capsid protein  
G01210\_P             -            260 Reo\_sigmaC           PF04582.7    326      0.21    9.4   6.7   3   3      0.67   1.4e+02    0.1   0.0    70   125   198   253   178   258 0.79 - Reovirus sigma C capsid protein  
G01210\_P             -            260 Syntaxin             PF00804.20   103       6.1    5.8   7.1   1   3         3   1.1e+03   -1.5   0.0    73    94    31    57    10    74 0.61 - Syntaxin  
G01210\_P             -            260 Syntaxin             PF00804.20   103       6.1    5.8   7.1   2   3     0.012       4.3    6.2   0.1     7    51    90   131    87   140 0.69 - Syntaxin  
G01210\_P             -            260 Syntaxin             PF00804.20   103       6.1    5.8   7.1   3   3      0.27        94    1.9   0.8    31    72   175   221   143   246 0.46 - Syntaxin  
G01210\_P             -            260 Syntaxin-6\_N         PF09177.6     97       1.1    8.3   5.7   1   2      0.04        27    3.9   0.4    37    64    91   118    53   140 0.48 - Syntaxin 6, N-terminal  
G01210\_P             -            260 Syntaxin-6\_N         PF09177.6     97       1.1    8.3   5.7   2   2    0.0063       4.2    6.5   0.5     9    62   202   256   186   258 0.90 - Syntaxin 6, N-terminal  
G01210\_P             -            260 THOC7                PF05615.8    139      0.13   11.2   9.4   1   2     0.014       7.5    5.5   0.3    99   133    90   124    86   128 0.80 - Tho complex subunit 7  
G01210\_P             -            260 THOC7                PF05615.8    139      0.13   11.2   9.4   2   2    0.0023       1.2    8.0   5.6    24   138   122   249   120   250 0.78 - Tho complex subunit 7  
G01210\_P             -            260 Tropomyosin          PF00261.15   237       7.4    4.2  11.1   1   3      0.39        93    0.6   0.0    30    73    19    62     5    80 0.72 - Tropomyosin  
G01210\_P             -            260 Tropomyosin          PF00261.15   237       7.4    4.2  11.1   2   3     0.033       7.8    4.2   0.1   193   233    96   136    89   140 0.81 - Tropomyosin  
G01210\_P             -            260 Tropomyosin          PF00261.15   237       7.4    4.2  11.1   3   3      0.11        27    2.4   4.3    13    83   185   256   178   259 0.82 - Tropomyosin  
G01210\_P             -            260 Vps5                 PF09325.5    236       1.6    6.6   5.8   1   2    0.0045       1.9    6.3   0.9   131   174    89   132     4   137 0.77 - Vps5 C terminal like  
G01210\_P             -            260 Vps5                 PF09325.5    236       1.6    6.6   5.8   2   2     0.081        34    2.2   0.3   166   203   201   234   178   252 0.65 - Vps5 C terminal like
